# Minimal SARS-CoV-2 Transmission After Implementation of a Comprehensive Mitigation Strategy at a School — New Jersey, August 20–November 27, 2020

**DOI:** 10.15585/mmwr.mm7011a2

**Published:** 2021-03-19

**Authors:** Kevin G. Volpp, Bruce H. Kraut, Smita Ghosh, John Neatherlin

**Affiliations:** ^1^Perelman School of Medicine and Wharton School of the University of Pennsylvania, Philadelphia, Pennsylvania; ^2^The Lawrenceville School, Lawrenceville, New Jersey; ^3^CDC COVID-19 Response Team.

During fall 2020, many U.S. kindergarten through grade 12 (K–12) schools closed campuses and instituted remote learning to limit in-school transmission of SARS-CoV-2, the virus that causes COVID-19 (*1*,*2*). A New Jersey grade 9–12 boarding school with 520 full-time resident students, 255 commuter students, and 405 faculty and staff members implemented a comprehensive mitigation strategy that included universal masking, testing, upgraded air-handling equipment to improve ventilation, physical distancing of ≥6 ft, contact tracing, and quarantine and isolation protocols to prevent and control transmission of SARS-CoV-2 among students, faculty, and staff members. Mandatory twice-weekly screening using real-time reverse transcription–polymerase chain reaction (RT-PCR) testing of all students and staff members during August 20–November 27, 2020, resulted in the testing of 21,449 specimens. A total of 19 (5%) of 405 faculty and staff members and eight (1%) of 775 students received positive test results; only two identified cases were plausibly caused by secondary transmission on campus. Comprehensive mitigation approaches including frequent testing and universal masking can help prevent outbreaks in in-person high school settings even when community transmission is ongoing.

During August 20–November 27, 2020, a private boarding school in New Jersey implemented rigorous, comprehensive strategies to prevent introduction and transmission of SARS-CoV-2, including requiring students to quarantine for 2 weeks before arriving, and, upon arrival, to provide documentation of a negative RT-PCR test result performed within 7–10 days before campus arrival (Box). Upon opening in the fall, the school conducted twice-weekly RT-PCR screening of all students, faculty, and staff members during August 20–November 27. Students’ specimens were tested by using Broad Laboratories anterior nasal swab, high-throughput version of the CDC 2019-nCoV RT-PCR Diagnostic Panel, validated in accordance with guidance by the College of American Pathologists (issued on March 19, 2020) and the Food and Drug Administration (issued on February 29, 2020).* Faculty and staff member saliva samples were processed by Accurate Diagnostic Laboratories^†^ (Salivary SARS-COV2 COVID-19 by RT-PCR) and could be collected without supervision. Anterior nasal swabs and saliva specimens were collected, stored, and processed according to the manufacturer’s Emergency Use Authorization (EUA) instructions. The interval between specimen collection and availability of results was 24–36 hours for students and 54–78 hours for faculty and staff members (inclusive of transit time). In addition, rapid antigen tests (Quidel Sofia SARS Antigen FIA)^§^ were administered per EUA instructions to test anyone who reported COVID-19–compatible symptoms.^¶^ A confirmed case was defined as a positive RT-PCR test result in any person (student, faculty, or staff member). Persons with a positive rapid antigen test result or symptoms consistent with COVID-19 while awaiting RT-PCR confirmation were immediately isolated either on campus in single rooms with an unshared bathroom or off campus under the supervision of a parent or guardian if a student. Students, faculty, and staff members with COVID-19–compatible symptoms and negative rapid antigen test results received confirmatory RT-PCR testing. Staff members who were trained and certified through the New Jersey Department of Health conducted case investigations and contact tracing. Initially, contacts of patients with confirmed COVID-19 were defined as persons with >10 minutes of continuous exposure within 6 ft of a person with COVID-19 during the 48 hours before testing. In November, this definition was changed to include persons with 15 minutes of cumulative exposure during the same timeframe (*3*).

BOX **Mitigation approaches used for SARS-CoV-2 prevention and control at a school — New Jersey, August 20–November 27, 2020**

**Establish prearrival protocol**

- Ask students and staff members to engage in a 2-week prearrival quarantine that includes physical distancing, mask-wearing when not at home, avoiding unnecessary travel, and refraining from indoor social gatherings.
- Provide arriving students with a mail-in SARS-CoV-2 testing kit.*
- Require proof of a negative RT-PCR^†^ test result.
- Delay arrival for students with confirmed positive test results.
- Establish on-campus 10-day quarantine for out-of-state and international students until they have three negative test results 3 days apart.

**Implement classroom safety measures**

- Require universal masking inside classrooms and classroom buildings.
- Maintain physical distancing: seat students at least 6 ft apart in classrooms.
- Limit number of students on campus by having a subset of students participate virtually on a rotating basis (approximately one third of the time).
- Equip classrooms, dining pick-up areas, and bathrooms with HEPA filters, and MERV 13 filters in air handling equipment throughout campus.

**Maintain physical distance outside of the classroom**

- Require universal masking outside of student dormitory rooms except during distanced dining and during supervised outdoor athletics.
- Limit team practices and suspend competition with other schools.
- Provide single rooms for boarding students and prohibit visitation in dormitory rooms.

**Develop testing and screening protocols**

- Monitor symptoms daily and check temperature twice daily.
- Have students collect anterior nasal swabs under clinical supervision for RT-PCR testing twice weekly.
- Arrange for faculty and staff members to self-collect saliva specimens for RT-PCR testing twice weekly.
- Administer rapid antigen tests^†^ at any time for anyone with COVID-19–compatible symptoms.^§^
- Confirm any antigen test results in symptomatic persons with RT-PCR.

**Implement innovative contact tracing tools and robust quarantine measures**

- Issue proximity tracing devices to be worn at all time on campus, except in dorm room, shower, or pool.
- Enforce CDC-recommended definition of a close contact.
- Use tracing system data to determine induration and proximity to identify close contacts.
- Provide single rooms for resident contacts and parental monitoring of at-home quarantine.

**Reinforce compliance with protocols: Best for All agreement** ^¶^

- Continue biweekly testing of contacts while in quarantine.
- Conduct educational webinars, conduct question-and-answer sessions with the leadership team on specific areas of the re-opening plan, send formal emails with linked resources, post important documents and updated frequently asked questions on the school’s website, and display omnipresent signage for all students, parents, faculty, and staff members.
- For students, conduct virtual school meetings, class and dormitory group sessions, and reminders of best practices through social media presentations (such as Tik-Tok) to reinforce the messages.
- Use a motivational contract for behavioral reinforcement and establish social norms around wearing masks and maintaining distance to protect one another.
- Implement a “strike” system to establish consequences for students who do not comply with mitigation measures and testing protocols.

**Abbreviations:** HEPA = high-efficiency particulate arrestance; MERV = minimum efficiency reporting value; RT-PCR = reverse transcription–polymerase chain reaction.

* Vault Test Kit, www.vaulthealth.com/covid

^†^ Quidel Sofia rapid antigen test, https://www.quidel.com/immunoassays/rapid-sars-tests/sofia-sars-antigen-fia

^§^ https://www.cdc.gov/coronavirus/2019-ncov/symptoms-testing/symptoms.html

^¶^ https://lawrenceville.myschoolapp.com/ftpimages/928/download/download_5262472.pdf

As part of the comprehensive mitigation strategy, all students, faculty, and staff members were asked to comply with a Best for All** agreement that reinforced personal responsibility for community well-being (Box). This agreement included maintaining a distance of 6 ft from others whenever feasible; wearing a mask in all shared community or public spaces; full participation in the testing, symptom tracking, and contact tracing protocols; hygiene protocol compliance; wearing a personal tracer device; and following rules about house and campus life regarding meals and dormitory visitation.

Students, faculty, and staff members were required to maintain 6 ft of distance whenever possible, cafeterias provided take-out service only, meals were eaten outdoors, and nonboarding students were not allowed in the residential dormitories. Classrooms, dining pick-up areas, and bathrooms were equipped with HEPA filters, and minimum efficiency reporting value (MERV) 13 filters were inserted in air handling equipment throughout campus. Athletic activities were conducted outdoors whenever possible, during which coaches remained masked at all times. Student participants were also required to be masked during athletic activities unless masking was not feasible because of the intensity of the aerobic activity. Indoor athletic activities required masking at all times by both coaches and players, except in the case of swimming, and the number of participants was kept to a minimum in the maximal space available for them to compete. No interscholastic competitions were allowed, and student participation in outside club sports was forbidden.

Although interscholastic athletics were cancelled, teams held daily practice sessions. Extracurricular activities also took place using similar approaches to those used in the classrooms (i.e., universal masking and physical distance of ≥6 ft). To support the identification of contacts, the school employed a Bluetooth-enabled personal tracer, “Peace of Mind” (POM),^††^ that persons were required to wear at all times on campus except while in the shower or in their rooms or the pool. This device, originally designed to be an emergency alerting system, was repurposed to enhance contact tracing efforts by collecting information on duration and proximity of interpersonal contact, thus providing contact tracers with objective data to aid with recall and help determine whether potential exposures were of sufficient risk to require quarantine (*3*). Persons identified as contacts were quarantined for 14 days and continued to be tested twice a week through the school’s screening program. Violations of rules included in the Best for All agreement would generate a “strike”; students who received three “strikes” were sent home and not allowed to attend in-person school for 2 weeks.

During August 20–November 27, RT-PCR tests were performed on 8,955 saliva specimens from 405 faculty and staff members and 12,494 nasal swab specimens from 775 students (Table). Overall, 17 (0.18%) faculty and staff member specimens and eight (0.06%) student specimens tested positive, representing 4% of faculty and staff members and 1% of students. An additional two faculty and staff members were tested outside of the school’s protocols and received positive test results off campus (Table). All persons whose screening test results were positive were asymptomatic at the time of testing. Among all persons with positive test results, five of 17 faculty and staff members and two of eight students reported mild symptoms after diagnosis; no one required hospitalization. A median of one faculty case (range = 0–4) and one student case (range = 0–1) was identified each week. Overall, 66 antigen tests were performed for 59 students and seven faculty and staff members with COVID-19–compatible symptoms; all results were negative.

**TABLE:** SARS-CoV-2 testing results and tracing of cases and contacts at a school — New Jersey, August 20–November 27, 2020

SARS-CoV-2 testing results	Faculty/Staff members (n = 405)	Students (n = 775)
**No. of specimens tested (average per person)**	8,955 (22.1)	12,494 (15.1)
**No. of RT-PCR–positive tests**	19*	8
Specimens tested, %	0.21	0.06
Persons receiving testing, %	4.7	1.0
**No. (%) of cases linked to on-campus transmission**	0 (—)	2 (25)^†^
**No. of contacts identified and quarantined**	17	14
**No. of contacts with positive test results**	0	0

**Abbreviation:** RT-PCR = reverse transcription–polymerase chain reaction.

* Two faculty or staff members with positive test results were linked to off-campus cases and are included for completeness of results.

^†^ No plausible off-campus source could be identified.

Case investigations suggested that the source of infection in 25 of 27 (93%) cases was likely off-campus contacts, including exposure to family members or friends with COVID-19 who lived off campus, external workplace exposures for spouses of faculty and staff members, or community exposures outside the school campus. For two boarding students with a new diagnosis of COVID-19, case investigators were unable to identify a likely off-campus source or find evidence of contact with persons on campus with COVID-19.

Contact tracing, based on reported duration of contact of within 6 ft of a person with COVID-19 aided by data from personal tracing devices, identified 14 school-based contacts of student patients and 17 school-based contacts of faculty and staff member patients. All contacts quarantined for 14 days, and none received a positive test result during quarantine, suggesting that the risk mitigation strategies put into place were effective in preventing transmission from patients to their contacts.

Overall, compliance with the Best for All agreement and student adherence to mitigation protocols were high. All staff members and faculty on campus were authorized to enforce the agreement through the observations of students as part of their regular daily duties, which served as reminders to students about the importance of ongoing compliance to reduce the risk for transmission from patients to contacts. Over the course of the semester, 10 (1.3%) of 775 students garnered three “strikes” and were sent home for 2 weeks.

## Discussion

A comprehensive mitigation strategy that included compulsory prearrival isolation and screening, twice-weekly SARS-CoV-2 testing, technology-enhanced contact tracing and quarantine, and an enforced behavioral agreement was effective in preventing in-school transmission of SARS-CoV-2 at a campus with substantial daily on- and off-campus interactions. During August–December 2020, many U.S. K–12 schools implemented fully online (12%) or hybrid models (58%) of instruction because of concerns about transmission in schools (*4*). In spring 2020 in Israel, a large SARS-CoV-2 outbreak in a high school was documented (*5*); however, a recent analysis of schools across Europe found relatively low levels of school-related transmission (*6*). Earlier in the U.S. pandemic, outbreaks were detected in camps at which campers did not wear masks and close contact occurred (*7*). Although risk for transmission has appeared lower in elementary schools, data about transmission in K–12 educational settings are lacking because of limited screening (*6*). Schools can minimize SARS-CoV-2 transmission during in-person learning if well-designed risk mitigation protocols are followed, including frequent facility-wide testing and universal masking.

Twice-weekly screening testing of the entire school population identified COVID-19 cases in eight students and 17 faculty and staff members over a 14-week period, which would approximate 74 cases per 100,000 for students and 300 per 100,000 for faculty and staff members during a 7-day period. During the same period, the county in which the school is located had a 7-day total incidence ranging from a low of 17 (late August through early September) to 402 (November 24) cases per 100,000 persons (*8*). Community testing did not include frequent, systematic testing of all persons and, for this reason, rates are not directly comparable. However, despite a substantial increase in the number of weekly cases in the county during this period^§§^ and the potential exposure of nonresident students, staff members, and faculty or their families to persons with cases in the community, the school did not experience any epidemiologically linked cases leading to clusters or outbreaks during this period. Whereas cases in the broader community were likely underascertained and the incidence underestimated, the ability of the boarding school to maintain a consistent incidence over time while the surrounding community experienced a substantial increase in cases suggests that the mitigation measures instituted by the school were effective. Similar to other school settings (*1*), the initial sources of infection for most identified cases were likely outside the school. Mandatory twice-weekly screening indicated minimal on-campus transmission, suggesting that routine testing and mitigation protocols focused on distancing and universal masking that are consistently implemented can succeed in reducing the likelihood of on-campus transmission (*8*).

For a campus to remain open, persons with newly identified cases should be rapidly isolated to reduce transmission; a strict regimen of physical distancing and universal masking is a necessary component of the comprehensive approach to preventing transmission of COVID-19. Among 27 persons with confirmed cases, 31 contacts were identified through the combined use of patient interviews and analysis of proximity data from tracing devices, which provided objective information that aided the inclusion and exclusion of contacts. Moreover, wearing POM devices highlighted the importance of complying with physical distancing guidelines for all students, faculty, and staff members. Although all contacts were required to quarantine, none received a positive test result, suggesting that adherence to the physical distancing protocols and mandated mask wearing was high among members of the school community. The contractual Best for All agreement, in which lack of compliance with protocols monitored by faculty and staff members resulted in disciplinary action for a few students, likely contributed in motivating students to adhere to the on-campus protocols. All the mitigation measures used could readily be applied to nonboarding schools or day camps.

The findings in this report are subject to at least two limitations. First, although most of the mitigation measures (masking, physical distancing, and hand hygiene) could be implemented at low cost, extensive screening and use of proximity devices for enhanced contact tracing activities might be less feasible in other settings because of cost. Setting up an extensive logistical operation to conduct twice weekly sample collection on campus of all students, faculty, and staff members with relatively fast turn-around-times was required, which also involved considerable expense. Second, the source of a given infection, especially if off campus, typically could not be independently verified; therefore, the estimate of the number of cases transmitted on campus might not be exact.

This investigation suggested that adherence to physical distancing, universal masking, and behavioral reinforcement in conjunction with improved air filtration, and frequent testing can be effective in preventing transmission within campus settings (*8*). As testing becomes more widely available, the findings from this study might help educators consider testing strategies for screening of students, faculty, and staff members to contain COVID-19 transmission while supporting in-person learning for high school students. Secondary schools, whether boarding or day schools or hybrids, can implement a combination of testing and mitigation strategies to help reduce transmission of SARS-CoV-2 at schools to support in-person learning. Comprehensive mitigation approaches including frequent testing and universal masking can help prevent outbreaks in in-person high school settings even when there is ongoing community transmission.

**Summary**

**What is already known about this topic?**

During fall 2020, many U.S. kindergarten through grade 12 (K–12) schools closed campuses and instituted remote learning because of concerns that significant in-school transmission of SARS-CoV-2 was not preventable.

**What is added by this report?**

Frequent facility-wide SARS-CoV-2 testing in a high school with both residential and commuter students was part of a comprehensive strategy, including universal masking, that reduced in-school SARS-CoV-2 transmission while allowing significant daily on- and off-campus movement. Of 19 cases among faculty and staff members and eight among students, two (7%) were considered to represent on-campus transmission.

**What are the implications for public health practice?**

Comprehensive mitigation approaches including frequent testing and universal masking can help prevent outbreaks in in-person high school settings even when community transmission is ongoing.

## Footnotes

*
https://www.fda.gov/medical-devices/coronavirus-disease-2019-covid-19-emergency-use-authorizations-medical-devices/vitro-diagnostics-euas

†
https://www.fda.gov/media/137773/download?utm_campaign=050820_PR_Coronavirus%20%28COVID9%29%20Update%3A%20Daily%20Roundup%20May%208%2C%202020&utm_medium=email&utm_source=Eloqua

§
https://www.quidel.com/immunoassays/rapid-sars-tests/sofia-sars-antigen-fia

¶
https://www.cdc.gov/coronavirus/2019-ncov/symptoms-testing/symptoms.html

**
https://lawrenceville.myschoolapp.com/ftpimages/928/download/download_5262472.pdf

††
https://pomtracer.com

§§
https://usafacts.org/visualizations/coronavirus-covid-19-spread-map/state/new-jersey/county/mercer-county

## References

[1] Sheen A, Ro G, Holani K, Pinheiro Dos Santos AC, Kagadkar F, Zeshan M. Impact of COVID-9-related school closures on children and adolescents worldwide: a literature review. J Am Acad Child Adolesc Psychiatry 2020;59:S253. 10.1016/j.jaac.2020.08.417

[2] Viner RM, Russell SJ, Croker H, School closure and management practices during coronavirus outbreaks including COVID-19: a rapid systematic review. Lancet Child Adolesc Health 2020;4:397–404. 10.1016/S2352-4642(20)30095-X 32272089 PMC7270629

[3] CDC. Coronavirus disease 2019 (COVID-19): interim guidance on developing a COVID-19 case investigation and contact tracing plan. Atlanta, GA: US Department of Health and Human Services, CDC; 2020. https://www.cdc.gov/coronavirus/2019-ncov/php/contact-tracing/contact-tracing-plan/overview.html

[4] Oster E. Beyond past due: data to guide US school reopenings. Nature 2021;589:8. 10.1038/d41586-020-03647-w 33402717

[5] Stein-Zamir C, Abramson N, Shoob H, A large COVID-19 outbreak in a high school 10 days after schools’ reopening, Israel, May 2020. Euro Surveill 2020;25:2001352. 10.2807/1560-7917.ES.2020.25.29.2001352 32720636 PMC7384285

[6] Ismail SA, Saliba V, Lopez Bernal J, Ramsay ME, Ladhani SN. SARS-CoV-2 infection and transmission in educational settings: a prospective, cross-sectional analysis of infection clusters and outbreaks in England. Lancet Infect Dis 2021;21:344–53. 10.1016/S1473-3099(20)30882-3 33306981 PMC7833602

[7] Szablewski CM, Chang KT, Brown MM, SARS-CoV-2 transmission and infection among attendees of an overnight camp—Georgia, June 2020. MMWR Morb Mortal Wkly Rep 2020;69:1023–5. 10.15585/mmwr.mm6931e1 32759921 PMC7454898

[8] Zimmerman KO, Akinboyo IC, Brookhart MA, ; ABC Science Collaborative. Incidence and secondary transmission of SARS-CoV-2 infections in schools. Pediatrics 2021;e2020048090. 10.1542/peds.2020-048090 PMC8015158 33419869

